# TRAIL and Paclitaxel Synergize to Kill U87 Cells and U87-Derived Stem-Like Cells *in Vitro*

**DOI:** 10.3390/ijms13079142

**Published:** 2012-07-20

**Authors:** Bo Qiu, Xiyang Sun, Dongyong Zhang, Yong Wang, Jun Tao, Shaowu Ou

**Affiliations:** 1 Department of Neurosurgery, First Hospital of China Medical University, No. 155, North Nanjing Street, Heping District, Shenyang 110001, Liaoning, China; E-Mails: fhxue2002@sohu.com (B.Q.); dongyong0622@hotmail.com (D.Z.); wangyongdl@126.com (Y.W.); cmu_taojun@yahoo.com (J.T.); 2 Department of Pharmaceutics, School of Pharmacy, Fudan University, 826 Zhangheng Road, Shanghai 201203, China; E-Mail: cmu_sunxiyang@yahoo.com

**Keywords:** glioma, glioma stem cells, U87-derived stem-like cells (U87-SLCs), tumor necrosis factor (TNF)-related apoptosis-inducing ligand (TRAIL), paclitaxel, apoptosis

## Abstract

U87-derived stem-like cells (U87-SLCs) were cultured using serum-free stem cell media and identified by both biological behaviors and markers. Tumor necrosis factor (TNF)-related apoptosis-inducing ligand (TRAIL) and paclitaxel (PX), in combination or alone, was used to treat U87-MG human glioma cells (U87 cells) or U87-SLCs. The results showed that TRAIL/PX cannot only synergistically inhibit U87 cells but also U87-SLCs. We observed a significantly higher apoptotic rate in U87 cells simultaneously treated with TRAIL/PX for 24 h compared to cells treated with either drug alone. Furthermore, there was a remarkably higher apoptosis rate in U87-SLCs induced by the TRAIL/PX combination compared with either drug alone. Unlike the simultaneous treatment in U87 cells, U87-SLCs were pretreated for 24 h with 1 μmol/L of PX followed by 1000 ng/mL of TRAIL. Protein assays revealed that TRAIL/PX synergy was related to DR4, cleaved caspase-8 and cleaved caspase-3 upregulation, whereas the mitochondrial pathway was not involved in TRAIL-induced apoptosis. The present study indicates that PX can sensitize U87 cells and U87-SLCs to TRAIL treatment through an extrinsic pathway of cell apoptosis. The combined treatment of TRAIL and PX may be a promising glioma chemotherapy because of its successful inhibition of U87-SLCs, which are hypothesized to influence chemotherapeutic outcomes of gliomas.

## 1. Introduction

Malignant gliomas, e.g., glioblastoma multiforme (GBM) and anaplastic astrocytomas (AA), are the most common and devastating primary brain tumors given their dismal prognosis [1,2]. Despite aggressive therapy with maximal safe surgical resection, radiation and chemotherapy, these tumors invariably are refractory to, or become resistant to, treatment and often recur [3,4]. The treatment of malignant gliomas is currently one of the most difficult challenges in oncology. Although cytoreductive surgery is always the primary treatment [5,6], adjuvant therapies, such as chemotherapy, have also been proven to be beneficial for managing malignant gliomas [7,8]. A single drug, however, cannot obtain optimal efficacy because tumor cells often exhibit major resistance to one type of chemotherapeutic drug [9]. Therefore, combined chemotherapy might be a rational and practical option.

Recent work suggests that the existence of glioma stem cells (GSCs) may be a critical factor influencing chemotherapeutic outcomes of gliomas [10–13]. According to the GSCs theory, gliomas develop as cellular and functional hierarchies originating from GSCs that are responsible for tumor initiation, maintenance, progression and recurrence [14–16]. GSCs possess unique survival mechanisms and distinct stem cell properties: the ability of self-renewal and differentiation, a marked ability to proliferate following a prolonged quiescent period, and enhanced expression of DNA repair genes. Consequently, GSCs can escape chemotherapy-induced cell death and remain dormant for extended periods after treatment but eventually re-enter the cell cycle, which leads to the recurrence of gliomas [10,13,17,18]. It is now hypothesized that GSCs are the major causes for intractability and therapeutic resistance, and gliomas will not be cured unless the GSCs are eliminated [11,12,19,20].

Tumor necrosis factor (TNF)-related apoptosis-inducing ligand (TRAIL) induces apoptosis in tumor cells by binding to death receptors TRAIL-R1/DR4 and TRAIL-R2/DR5. These receptors possess an intracellular death domain, which triggers the activation of the caspase signaling cascade with or without the involvement of mitochondria after receptor-ligand association [21]. TRAIL is the safest and the most promising death ligand for clinical application compared with other death ligands of the TNF-a family because of its relatively selective cytotoxic activity for cancer cells over normal cells [22]. Nevertheless, some studies have found that gliomas easily build resistance to TRAIL-based therapies [23,24]. Tumor cell resistance to TRAIL seems to occur through the modulation of various molecular targets, including differential expression of death receptors, constitutive activation of Akt and nuclear factor-κB (NF-κB), overexpression of antiapoptotic molecules, mutations in apoptotic genes, defects in caspase signaling, and caspase inhibition in resistant cells [25,26]. TRAIL may ultimately have greater efficacy when used in combination with traditional chemotherapy because chemotherapeutic agents can sensitize tumors to TRAIL-induced apoptosis via different mechanisms [23,27,28]. Paclitaxel (PX), a cytotoxic agent that promotes microtubule assembly, inhibits microtubule depolymerization and, as a result, blocks dividing cells at the G2/M portion of the cell cycle, has been investigated in some phase I and II studies for treating gliomas, although the therapy was of very limited value [29,30]. In previous studies, we and others reported that TRAIL and PX have cooperative effects on gliomas *in vitro* and *in vivo* [31,32], which indicates a possible synergic effect and a promising combinatorial chemotherapy regimen for gliomas. Although it was reported that GSCs were resistant to either TRAIL [33] or PX [13] in laboratory investigations, their combinatorial effects on GSCs have not yet been investigated.

In this study, we designed and conducted a series of assays to assess the combined effects of TRAIL/PX on both U87 cells and U87-SLCs, with a particular focus on the latter. Our results might help to model combined TRAIL/PX treatment in anti-glioma chemotherapy, to provide experimental support for screening drugs effectively controlling GSCs, and to better understand the intrinsic and extrinsic pathways of cell death that can sensitize tumors to TRAIL-induced apoptosis.

## 2. Results and Discussion

### 2.1. Culture and Identification of Cells

**Figure 1:**
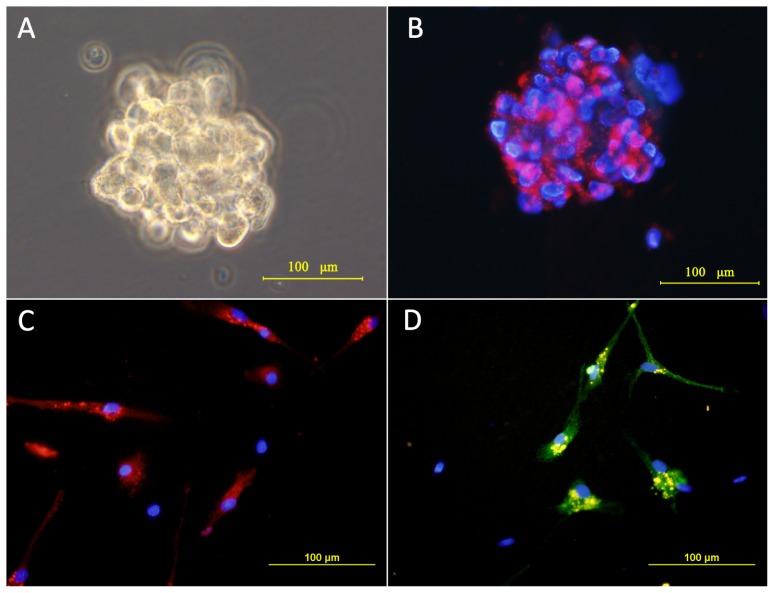
(**A**) Tumor spheres cultured in serum-free stem cell media (magnification, 200×); (**B**) Tumor sphere cells were CD133-positive (magnification, 200×); (**C**) and (**D**) Tumor spheres differentiated to express the glial cell marker, glial fibrillary acidic protein (GFAP) (red) and neuronal marker, Tu-20 (green). Cell nuclei were counterstained with 4, 6-diamidino-2-phenylindole (DAPI; blue, magnification, 200×).

U87 cells were cultured as monolayers and passaged in FBS-containing media. When switched into the stem cell media, single cell division occurred in 3 days, followed by the formation of large numbers of “neurosphere-like” tumor spheres within 6–7 days, which contained approximately 4–8 cells per sphere. Growth was slow in the first few weeks, but within 2 weeks, the majority of the spheres had increased their diameters by 5–10-fold. After approximately 2 weeks of culture, the formation of tumor spheres was observed and imaged under a phase-contrast microscope (Figure 1A).

To validate their proliferative ability, tumor spheres were dissociated into a single-cell suspension and passaged at a ratio of 1:2 or 1:3. Cell cleavage occurred in 2 days, and new tumor spheres formed within 1 week. Serial passage revealed that the tumor sphere cells maintained proliferation ability after at least 4 generations. In a limited dilution assay, the single cells from the tumor spheres were serially diluted and reseeded in microwells. After quantifying by microscope, it was determined that more than 50% of the single cells in the microwells were capable of forming secondary tumor spheres, although their diameters were generally less than those of the primary spheres. This assay demonstrated the self-renewing properties of the tumor sphere cells.

The tumor spheres were immunostained for CD133, the committed marker of GSCs [14–16]. The majority of the tumor sphere cells were CD133-positive concomitantly with the plasma membrane, as shown in Figure 1B. Upon exposure to FBS-containing media, the tumor spheres became flat, and the cells began to migrate out from the tumor spheres. After seven days, they stained positive for either beta-tubulin III (a neuronal marker) or for glial fibrillary acidic protein (GFAP; a glial cell marker) (Figure 1C,D). This observation is consistent with previous reports [14–16], which showed CD133-positive multipotent GSCs differentiated into neurons and glia. Therefore, U87-SLCs were identified by both biological behaviors and markers.

### 2.2. Cell Cycle Analysis

**Figure 2:**
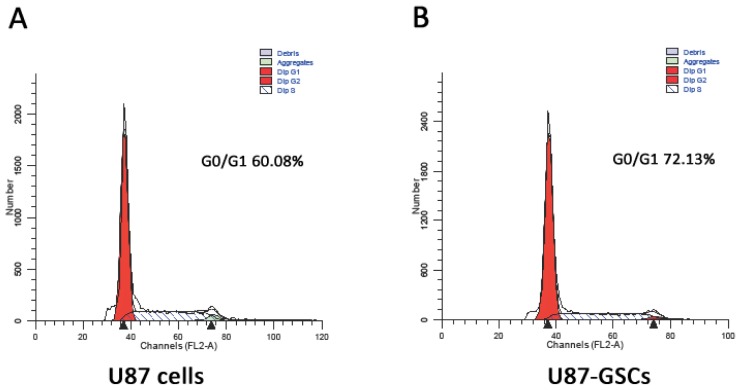
The proportion of cells in G0/G1 phase. (**A**) U87 cells; (**B**) U87-SLCs.

As depicted in Figure 2, cell cycle analysis demonstrated that the proportion of U87-SLCs in stationary phase was obviously higher than that of U87 cells (72.13% *vs.* 60.08%, *p* < 0.05). The results indicate that most U87-SLCs are quiescent, which is concordant with the findings of other studies [3,17,34,35].

### 2.3. Chemotherapeutic Effects of TRAIL and PX on U87 Cells

**Figure 3:**
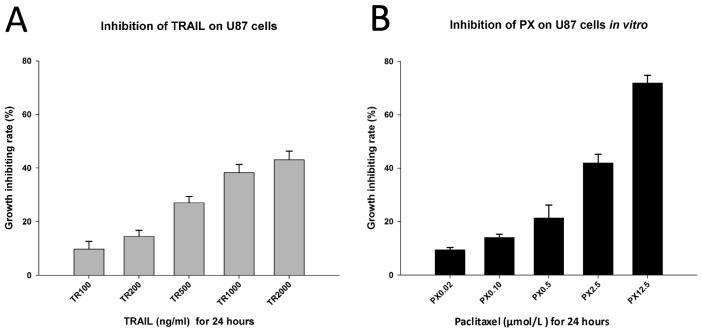

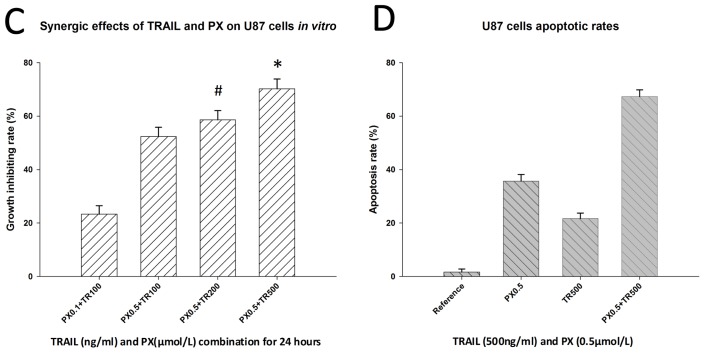
(**A**) and (**B**) In an MTT assay, TRAIL or PX alone inhibited U87 cells *in vitro* in a concentration-dependent manner; (**C**) The synergic effect of TRAIL/PX on U87 cells (# CDI = 0.83; * CDI = 0.59); (**D**) Flow cytometric apoptosis assay.

As expected, both TRAIL and PX demonstrated an inhibitory effect on U87 cells *in vitro* in MTT assay. We observed that TRAIL inhibited U87 cells *in vitro* when its concentration was more than 100 ng/mL, and the inhibiting effect was concentration-dependent. Nevertheless, there was no significant difference in the growth-inhibiting rate when the concentration of TRAIL reached 1000 ng/mL or 2000 ng/mL, suggesting that TRAIL has achieved its saturated concentration at 1000 ng/mL (Figure 3A). PX also exhibited an inhibiting effect on U87 cells *in vitro* in a concentration-dependent manner. PX inhibited U87 cell growth *in vitro* when its concentration was 0.02 μmol/L, and the growth-inhibiting rate increased with higher concentrations. When the PX concentration was increased to 12.5 μmol/L, its growth-inhibiting rate was over 70% (Figure 3B).

The synergic effect of both drugs was subsequently analyzed by MTT assay. After 24 h of incubation, the absorbance of each group was measured and calculated according to the committed equations. Combined TRAIL/PX showed a synergic effect on U87 cells when their concentrations were 200 ng/mL and 0.5 μmol/L, respectively (coefficient of drug interaction (CDI) = 0.83). A significant synergistic effect was observed when their concentrations were 500 ng/mL and 0.5 μmol/L, respectively (CDI = 0.59, Figure 3C). Nevertheless, increasing the concentrations of TRAIL or PX did not yield statistically significant different CDI values. Moreover, the drug concentrations of TRAIL and PX were much lower than when used alone, which would reduce their cytotoxicity. These results were in accordance with findings of previous studies [31,32].

Using a flow cytometric apoptosis assay, a significantly higher apoptotic rate was observed with the combined treatment of TRAIL (500 ng/mL) and PX (0.5 μmol/L) for 24 h than with either drug alone (*p* < 0.001, Figure 3D).

### 2.4. Inhibitory and Apoptosis-Inducing Effects of TRAIL and PX on U87-SLCs

**Figure 4:**
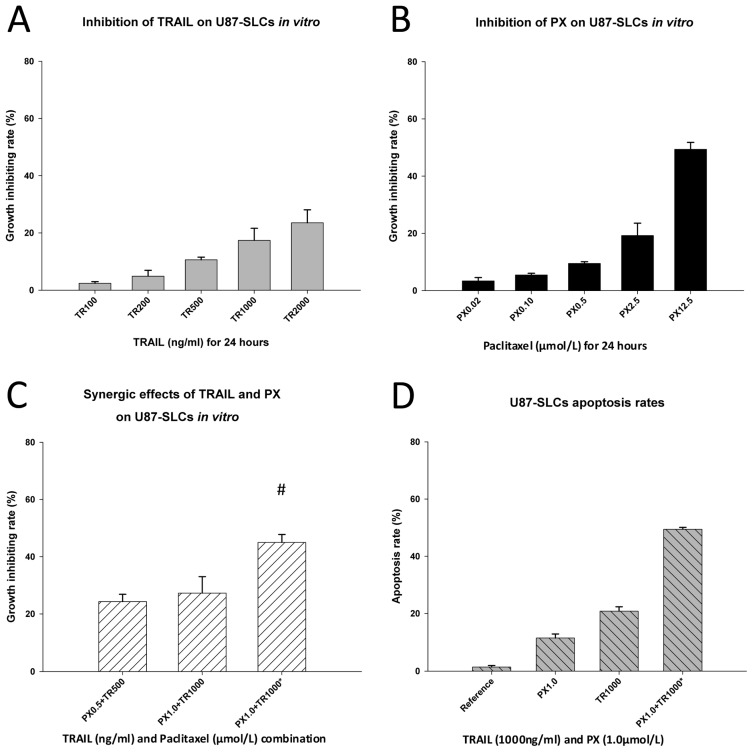
(**A**) TRAIL weakly inhibited U87-SLCs; (**B**) A high concentration of PX showed a relatively high inhibitory effect on U87-SLCs; (**C**) No synergy occurred when U87-SLCs were treated simultaneously with TRAIL and PX after 24 h. However, a modified administration scheme was synergic (# CDI = 0.80); (**D**) Flow cytometric apoptosis assay.

By contrast, the effects of TRAIL or PX alone on U87-SLCs were remarkably weak. Incubation of TRAIL at different concentrations for 24 h only slightly inhibited U87-SLCs. The growth-inhibiting rate was merely 23.5% even at a concentration of 2000 ng/mL (Figure 4A), whereas high concentrations of PX showed a relatively higher inhibitory effect on U87-SLCs after 24 h. At a concentration of 12.5 μmol/L, the growth-inhibitory rate of U87-SLCs was 49.4% (Figure 4B). These results corroborate the theory that GSCs are resistant to concurrent chemotherapeutics [11,13,20,36,37]. The chemoresistance of GSCs may result from their stem cell characteristics and a higher expression of ATP-binding cassette transporter protein, MGMT (O6-methylguanine-DNA methyltransferase), drug efflux pumps, anti-apoptosis proteins, and inhibitors of apoptosis protein families [13,38–40]. Additionally, chemoresistance of GSCs to TRAIL or PX has been found and reported elsewhere [13,33,36] and was confirmed in our study.

An exciting result occurred when both drugs were combined and incubated with U87-SLCs. Initially, both drugs at different concentrations were used to simultaneously treat cells for 24 h, but no synergic effects were found. Subsequently, the administration time was adjusted. We found that a 24 h pretreatment of 1 μmol/L of PX followed by 1000 ng/mL of TRAIL for an additional 24 h period produced a synergic effect and a CDI of 0.80 (Figure 4C).

Apoptosis was evident 24 h after the concentrations of TRAIL and PX described above were added to U87-SLCs at different time points, and the apoptotic rate was significantly higher than using either drug alone (*p* < 0.001, Figure 4D). Pretreatment with PX sensitized U87-SLCs to TRAIL-mediated apoptosis. This result is consistent with the findings of other studies that showed renal cancer cells [41] or small cell lung cancer cells [42] were also sensitized by PX to TRAIL-induced apoptosis. Moreover, the required dose of each drug is lower in combination than when used alone, thus the effects of combined treatment were less cytotoxic.

### 2.5. TRAIL/PX Synergy Occurs through Upregulation of DR4, Caspase-8 and Caspase-3

As reported, tumor cell sensitization by PX may be related to greater caspase activation, abrogation of the mitotic checkpoint, and upregulation of DR4 and DR5 receptors [28,43,44]. In the current study, we determined several proteins related to the extrinsic and intrinsic pathways of cell apoptosis.

In U87 cells, the expression of DR4 increased after exposure to TRAIL/PX compared with the expression of DR4 after exposure to each drug alone. In U87-SLCs, the expression of DR4 was significantly increased after exposure to the combined TRAIL/PX treatment. DR5 expression, however, showed no increase when treated with TRAIL/PX, whether in U87 cells or U87-SLCs. These results suggest that TRAIL/PX synergy might be related to enhanced expression of death receptors DR4 but not DR5.

**Figure 5:**
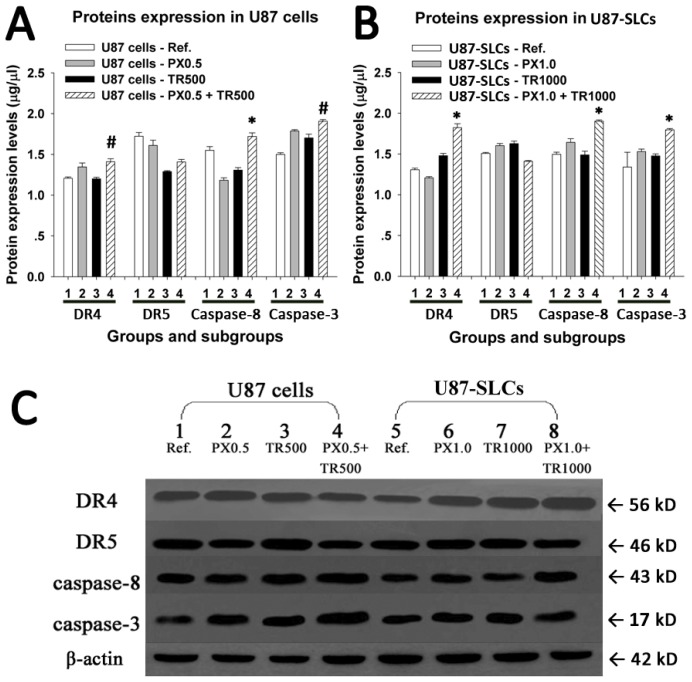
(**A**) and (**B**) In the TRAIL/PX combination treatment with synergy, significantly higher expression levels of DR4, cleaved caspase-8 and cleaved caspase-3, but not DR5, were observed by quantitative analysis (# *p* < 0.05; * *p* < 0.01) either in U87 cells or U87-SLCs; (**C**) Upregulation of DR4, cleaved caspase-8 and cleaved caspase-3 in the TRAIL/PX combination treatment with synergy was visualized by Western blot.

To further evaluate downstream activation of apoptosis signaling following increased expression of the death receptor DR4, caspase-8 and caspase-3 expression were characterized. A significant increase in cleaved caspase-8 was found in both U87 cells and U87-SLCs after the TRAIL/PX treatment compared with each treatment alone. Similarly, cleaved caspase-3 was significantly elevated in both U87 cells and U87-SLCs when exposed to TRAIL/PX. We evaluated whether the production of cleaved caspase-3 after the TRAIL/PX treatment occurred through the intrinsic apoptotic pathway by quantifying the expression of cytochrome c. Cytochrome c expression was not elevated in either U87 cells or U87-SLCs or even decreased in U87 cells (data not shown). These results indicated the mitochondrial pathway may not be involved in the apoptosis of glioma cells tested with our experimental parameters. The results are shown in Figure 5.

Based on the experimental data, we speculate that the apoptosis induced by the TRAIL/PX combination is related to the upregulated DR4 expression and caspase activation through an extrinsic pathway and that the intrinsic or mitochondrial pathway is not utilized. These findings suggest the mitochondrial pathway might not be necessary for TRAIL-induced apoptosis because it can be bypassed by direct caspase-8-mediated activation of downstream caspases [45,46]. However, the combined effects of TRAIL/PX might be more complicated than a simple gradual elevation of proapoptotic signals, and the mechanisms are to be further investigated in future studies.

## 3. Experimental Section

### 3.1. Cell Culture

U87-MG human glioma cell lines were obtained from American Type Culture Collection (ATCC, Bethesda, MD, USA). U87 cells of low passage were grown and maintained in Dulbecco’s modified Eagle media (DMEM)/F-12 (Gibco, Carlsbad, CA, USA) containing 10% fetal bovine serum (FBS, Hyclone, Logan, UT, USA) and antibiotics Penicillin G (100 IU/mL) and streptomycin (100 μg/mL) at a density of 1 × 10^6^ cells/mL. The cells were grown in a standard tissue culture incubator with 5% CO_2_ in humidified air at 37 °C. During the logarithmic growth phase (log phase), some cells were dissociated using 0.1% trypsin (Invitrogen, Carlsbad, CA, USA) and suspended in stem cell media at a density of 1 × 10^6^ cells/mL, and the other cells were still cultured in serum-containing medium and passaged with trypsin digestion. The stem cell media was serum-free DMEM/F-12 supplemented with B-27 (Invitrogen), 20 ng/mL recombined human epidermal growth factor (rhEGF, Invitrogen), 20 ng/mL basic recombined human fibroblast growth factor (rhFGF-b, Invitrogen), 100 IU/mL Penicillin G and 100 μg/mL Streptomycin. Fresh EGF and FGF were added to the media each week, and the serum-free media was changed twice a week. When the spheres emerged, they were passaged by trituration through a fire-narrowed Pasteur pipette and reseeded into stem cell media at the same density to form subspheres. To evaluate the self-renewal capacity of tumor spheres, the single-cell suspensions were reseeded into 96-well microwell plates by limited dilution at a cell density of 1–2 live cells per well. Each well was fed with fresh stem cell media every 2 days. The tumor spheres were inspected and imaged under a phase-contrast microscope (IX70; Olympus, Tokyo, Japan).

### 3.2. Induced Differentiation of Tumor Sphere Cells

A differentiation assay was carried out to assess the multipotency of the tumor spheres cells. The tumor spheres were harvested and cultured on coverslips precoated with poly-L-lysine (Sigma, St. Louis, MO, USA) in DMEM/F-12 containing 10% FBS in individual wells of a 24-well culture plate. The cells were fed with FBS-supplemented media every 2 days, and the coverslips were processed 7 days later using immunocytochemistry.

### 3.3. Immunocytochemistry of Cultured Cells

The cultured tumor spheres and their differentiated progeny cells were subjected to an immunocytochemistry assay to detect lineage-specific markers described previously [14–16,47]. In brief, the tumor spheres were collected and plated onto anti-peeling slides (Corning, NY, USA) and incubated in stem cell media for 4 h. The cells were then fixed in 4% paraformaldehyde and incubated at 4 °C with a primary mouse monoclonal antibody against CD133 (1:200, Abcam, Cambridge, UK) in accordance with the manufacturer’s protocol. After 24 h, Cy3-conjugated goat anti-mouse secondary antibody (1:50, Sigma) was then added and incubated for 2 h at room temperature.

Immunostaining of differentiated tumor cells was performed 7 days after induced differentiation. The cells were fixed in 4% paraformaldehyde and then incubated with primary antibodies against GFAP (monoclonal rabbit anti-GFAP, 1:200, Chemicon International, Temecula, CA, USA) for glial cells and TU-20 (monoclonal mouse anti-beta-tubulin III isoform, *C*-terminus, 1:200, Millipore, Billerica, MA, USA) for neurons at 4 °C for 24 h. Cy3-conjugated secondary antibody (goat anti-rabbit, 1:250, Sigma) and fluorescein isothiocyanate (FITC)-conjugated secondary antibody (goat anti-mouse, 1:250, Sigma) were added and incubated for 2 h at room temperature.

The cells were counterstained with DAPI (Invitrogen) to identify nuclei. In control samples, the primary antibody was replaced by isotype IgG. A fluorescent microscope (BX61, Olympus, Tokyo, Japan) was used to observe and image the cells.

### 3.4. Flow Cytometric Analysis of the Cell Cycle

Briefly, U87 cells or U87-derived tumor sphere cells (1 × 10^6^ cells/mL) were maintained under different culture conditions for 24 h and then all the cells were harvested, dissociated, and washed with 2 mL phosphate buffered saline (PBS). The cells then were fixed at 4 °C with cold 70% ethanol for at least 30 min and stored at −20 °C overnight. Ethanol was removed by centrifugation, and 2 mL of PBS was added to wash the pellets. The cellular DNA was stained with propidium iodide (PI, 50 μg/mL, Sigma) and RNAse (100 μg/mL, Sigma) for 30 min. The DNA content data of the stained cells were acquired on BD FACS Calibur and analyzed with CellQuest software (Becton–Dickinson, Franklin Lakes, NJ, USA). Experiments were performed in triplicate.

### 3.5. Respective and Synergic Effects of TRAIL and PX on U87 Cells

U87 cells were divided into a reference group, a PX group, a TRAIL (TR) group, and a combination group. The cells in log phase were plated in 96-well plates (5 × 10^4^ cells per well in a volume of 100 μL). The cells were cultured for 24 h and then treated with media containing different concentrations of drugs, 100 μL per well and 4 parallel wells for each concentration. After an additional 24-h culture period, 10 μL of 3-(4,5)-dimethylthiazol-2-yl)-2,5-diphenyl tetrazolium bromide (MTT, Sigma) solution (5 mg/mL) was added to each well and incubated at 37 °C for an additional 4 h. After aspirating solution from each well, 100 μL of dimethyl-sulfoxide (DMSO, Sigma) solution was added into each well to dissolve the crystals. The absorbance (Abs) of the individual wells was detected at 570 nm with a Microplate Reader (Bio-Rad, Hercules, CA, USA). The growth-inhibiting rate of the cells was determined by the following Equation 1:

(1) $$\text{Growth}-\text{inhibiting rate }(\%)=(1-\text{Abs}{\;}_{\text{test cells}}/\text{Abs}{\;}_{\text{reference cells}})\times 100\%$$

Experiments were performed in triplicate. In MTT assay, the coefficient of drug interaction (CDI) was used to analyze the effects of drug combination [48], which is calculated as follows (2):

(2) CDI=AB/(A×B)

According to the absorbance of each group, AB is the ratio of the combination groups to reference group and *A* or *B* is the ratio of the single agent group to reference group. Thus, a CDI less than 0.85 indicate that the drugs are synergistic, and a CDI less than 0.75 indicate that the drugs are significantly synergistic.

An apoptosis assay using flow cytometry was performed to detect U87 cells apoptotic rates. After 24 h of incubation with the drugs of different concentrations, as described above, the cells were trypsinized and collected with the supernatant. The cells were then washed in cold PBS, adjusted to 1 × 10^6^ cells/mL, labeled with annexin V-FITC and PI (Annexin V-FITC Kit, BD, San Diego, CA, USA), and analyzed with a FACScan flow cytometer (BD, San Jose, CA, USA). The treatments were performed in triplicate, and the percentage of labeled cells undergoing apoptosis in each group was determined and calculated.

### 3.6. Respective and Synergic Effects of TRAIL and PX on U87-SLCs Cells

The procedures were the same as in Section 3.5. U87-SLCs cultured in stem cell media were divided into a reference group, a PX group, a TRAIL (TR) group, and a combination group. The cells were then processed as described previously in Section 3.5. The equations for the growth-inhibiting rate or the CDI were the same as described above. An apoptosis assay was also performed to determine the U87-SLCs apoptotic rates after drug treatment, and the procedure was the same as described in Section 3.5.

### 3.7. Assessment of Proteins Related to TRAIL-Induced Apoptosis

Several proteins related to TRAIL-induced apoptosis were analyzed, including DR4, DR5, caspase-8, caspase-3, and cytochrome *c*. After washing, all proteins from detergent-lysed cells were quantified using a bicinchoninic acid (BCA) protein assay kit (R & D Systems, Minneapolis, MN, USA) according to the manufacturer’s instructions. Sodium dodecyl sulfate polyacrylamide gel electrophoresis (SDS/PAGE) and Western blot analyses were then performed as described in a previous study [49]. Fifty microgram of protein from each group was resolved by SDS-PAGE, transferred onto polyvinylidene fluoride (PVDF) membranes (Roche, Indianapolis, IN, USA) by electroblotting, probed with specific primary antibodies, followed by secondary antibodies conjugation, and analyzed. The primary antibodies were monoclonal mouse anti-human DR4 (1:200, Santa Cruz, CA, USA), monoclonal mouse anti-human DR5 (1:200, Santa Cruz), monoclonal mouse anti-human caspase-3 (1:1000, R & D Systems), monoclonal mouse anti-human caspase-8 (1:500, R & D Systems), and monoclonal mouse anti-human cytochrome c (1:50, BD Pharmingen, San Diego, CA, USA). The primary antibodies were detected using horseradish peroxidase-conjugated goat anti-mouse IgG (1:5000, Promega, Heidelberg, Germany) in blocking solution. Immunoreactive protein bands were detected with enhanced chemiluminescence reagent (ECL-PLUS) and densitometrically quantitated according to the manufacturer’s instructions (Amersham Pharmacia Biotech, Piscataway, NJ, USA).

### 3.8. Statistical Analysis

All experiments were performed at least three times, and the representative results are presented as the mean ± standard deviation. Statistical comparisons were made with Student’s *t*-test. The data were analyzed and visualized using SigmaPlot 11.0 software (Systat Software, Inc. Chicago, USA).

## 4. Conclusions

The data obtained in the present study indicate that the TRAIL/PX combination inhibits growth and induces apoptosis more than either agent alone in both U87 cells and U87-SLCs. PX may sensitize U87 cells and U87-SLCs to TRAIL-induced apoptosis by upregulating DR4, caspase-8 and caspase-3 through an extrinsic pathway. This combinatorial regimen is expected to be a promising adjuvant therapy for gliomas in the future. Further investigation is needed to establish an optimal strategy and to delineate the mechanism more precisely. Only one glioma cell line was used in this study; therefore, more studies are necessary to strengthen our conclusions. Extensive work should be performed in GSC lines from recent glioma patient tissues to determine the cell reaction to combined TRAIL/PX therapy, both *in vitro* and *in vivo*.
